# Root system architecture associated zinc variability in wheat (*Triticum aestivum* L.)

**DOI:** 10.1038/s41598-024-52338-3

**Published:** 2024-01-20

**Authors:** Mehwish Noor, Aysha Kiran, Muhammad Shahbaz, Muhammad Sanaullah, Abdul Wakeel

**Affiliations:** 1 Department of Botany, University of Agriculture, Faisalabad, 38040 Pakistan; 2 Institute of Soil and Environmental Sciences, University of Agriculture, Faisalabad, 38040 Pakistan

**Keywords:** Imaging, Nutrition, Quality of life, Biochemistry, Metals, Plant breeding, Plant physiology, Plant stress responses, Natural variation in plants

## Abstract

Root system architecture (RSA) plays a fundamental role in nutrient uptake, including zinc (Zn). Wheat grains are inheritably low in Zn. As Zn is an essential nutrient for plants, improving its uptake will not only improve their growth and yield but also the nutritional quality of staple grains. A rhizobox study followed by a pot study was conducted to evaluate Zn variability with respect to RSA and its impact on grain Zn concentration. The grain Zn content of one hundred wheat varieties was determined and grown in rhizoboxes with differential Zn (no Zn and 0.05 mg L^−1^ ZnSO_4_). Seedlings were harvested 12 days after sowing, and root images were taken and analyzed by SmartRoot software. Using principal component analysis, twelve varieties were screened out based on vigorous and weaker RSA with high and low grain Zn content. The screened varieties were grown in pots with (11 mg ZnSO_4_ kg^−1^ soil) and without Zn application to the soil. Zinc translocation, localization, and agronomic parameters were recorded after harvesting at maturity. In the rhizobox experiment, 4% and 8% varieties showed higher grain Zn content with vigorous and weaker RSA, respectively, while 45% and 43% varieties had lower grain Zn content with vigorous and weaker RSA. However, the pot experiment revealed that varieties with vigorous root system led to higher grain yield, though the grain Zn concentration were variable, while all varieties with weaker root system had lower yield as well as grain Zn concentration. Zincol-16 revealed the highest Zn concentration (28.07 mg kg^−1^) and grain weight (47.9 g). Comparatively higher level of Zn was localized in the aleurone layer than in the embryonic region and endosperm. It is concluded that genetic variability exists among wheat varieties for RSA and grain Zn content, with a significant correlation. Therefore, RSA attributes are promising targets for the Zn biofortification breeding program. However, Zn localization in endosperm needs to be further investigated to achieve the goal of reducing Zn malnutrition.

## Introduction

Zinc (Zn) is a vital micronutrient for both plants and humans, as it is involved in DNA and protein synthesis, cell growth, supporting the immune system, and maintaining good health^1^. According to the World Health Organization (WHO), globally, 2 billion people are suffering from “hidden hunger” due to malnutrition of trace elements, specifically Zn. Approximately 450,000 children under the age of five are suffering from zinc deficiency^2^. Zinc is an important microelement, its deficiency is widespread, specifically in developing nations such as Pakistan and India^3^. Zinc deficiency is most common in cereal crops, especially wheat. Higher intake of wheat products in many developing countries is a major reason for Zn malnutrition because wheat is inherently low in Zn content and high in phytate, which further limits Zn bioavailability^4^. Processing of wheat also reduces the intake of Zn in humans, as the wheat bran and aleurone layers are usually lost during refining^5^. Micronutrient deficiency reduces nutritive properties and crop production, which ultimately affects human health^6^. To increase wheat yield and quality, a balanced application of nutrients must be applied at the right time, in the right amount, and in the right place^7^.

Availability of Zn in the rhizosphere and plant genotype are two main factors responsible for low Zn in wheat grain. Zinc uptake by the root is the first step in the movement of Zn from soil to grain^8^. Rose et al. (2013) suggested that more root surface area and enhanced root length density improve the uptake of immobile Zn^9,10^. Generally, Zn deficiency is associated with the seedling’s development stage of plant growth at 15–20 days after seedling emergence^11^. To enhance nutrient use efficiency in the plant body, there is a need to explore RSA complexity and inclusion in breeding approaches^12^. Root exudation can also lead to enhance Zn uptake by plants^13^.

Increased daily intake of Zn through wheat-processed diets is an important way to overcome Zn deficiency. Currently, Zn concentration in wheat grain is 31.84 mg kg^−1^, which does not fulfill the recommended Estimated Average Requirement (EAR) of Zn, i.e., 10.3 mg d^−1^^14^. Zinc biofortification of wheat edible parts such as grain/endosperm is main target of the current research needs^15^. It is an effective strategy to deal with Zn malnutrition in developing countries. Furthermore, application of Zn fertilizer to soil reduces the heavy metal uptake enhancing Zn concentration in grain^16,17^. Along with enhancing Zn in grain, suitable processing, and food forms of wheat are crucial to meeting human Zn needs through wheat. Zinc enriched variety Zincol-16 was compared with wheat cultivar Galaxy-13 for improvement of Zn in the human population in Pakistan under the BiZiFED project. The findings of this project demonstrated that consumption of processed biofortified wheat resulted in a 1.5 mg per day (22% increase) in total dietary Zn intake in consumers as compared with a control variety relying on staple foods^18^.

It was hypothesized that RSA significantly contributes to Zn absorption by wheat leading to enhanced grain Zn content. Therefore, it is worth working to identify the most Zn-responsive wheat varieties by characterizing the RSA role in Zn uptake and its translocation from roots to shoot to grain. It will reduce Zn malnutrition in masses if processed carefully.

## Results

In the current study, 100 varieties of wheat were screened for their grain Zn content and evaluated for Zn association with root system architecture attributes. Further, selected varieties were evaluated for Zn uptake, translocation, concentration, and localization in the grain.

### Study I: Association of root system architecture with grain Zn content in a rhizobox based system

#### Grain Zn concentration and genetic variation

**Figure 1:**
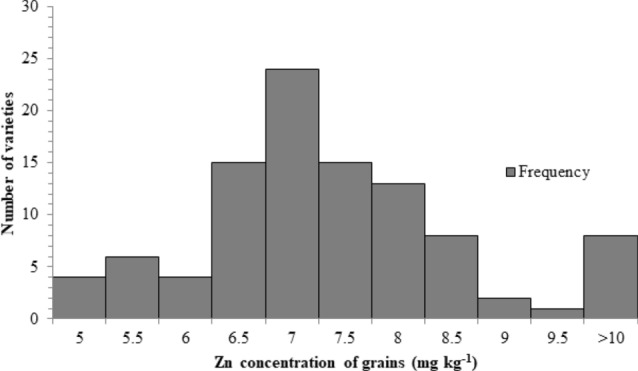
Frequency distribution histogram representing 100 wheat varieties grain Zn concentration. Displaying counts of categorical quantitative single variable. Where columns correspond to bins that together span the range of the data, height of each bar corresponding to the number of wheat varieties falling into each bin.

Large variations in grain Zn contents were observed in these 100 wheat varieties, ranging from 4 to 23 mg kg^−1^. The frequency distribution for grain Zn concentration is presented in a histogram (Fig. 1). The distributional peak is towards the right-side limits from the center, and the tail stretches away from the center. The highest number of varieties have grain Zn content within the range of 7 mg kg^−1^ (Fig. 1).

### Genetic variation in RSA attributes

**Figure 2:**
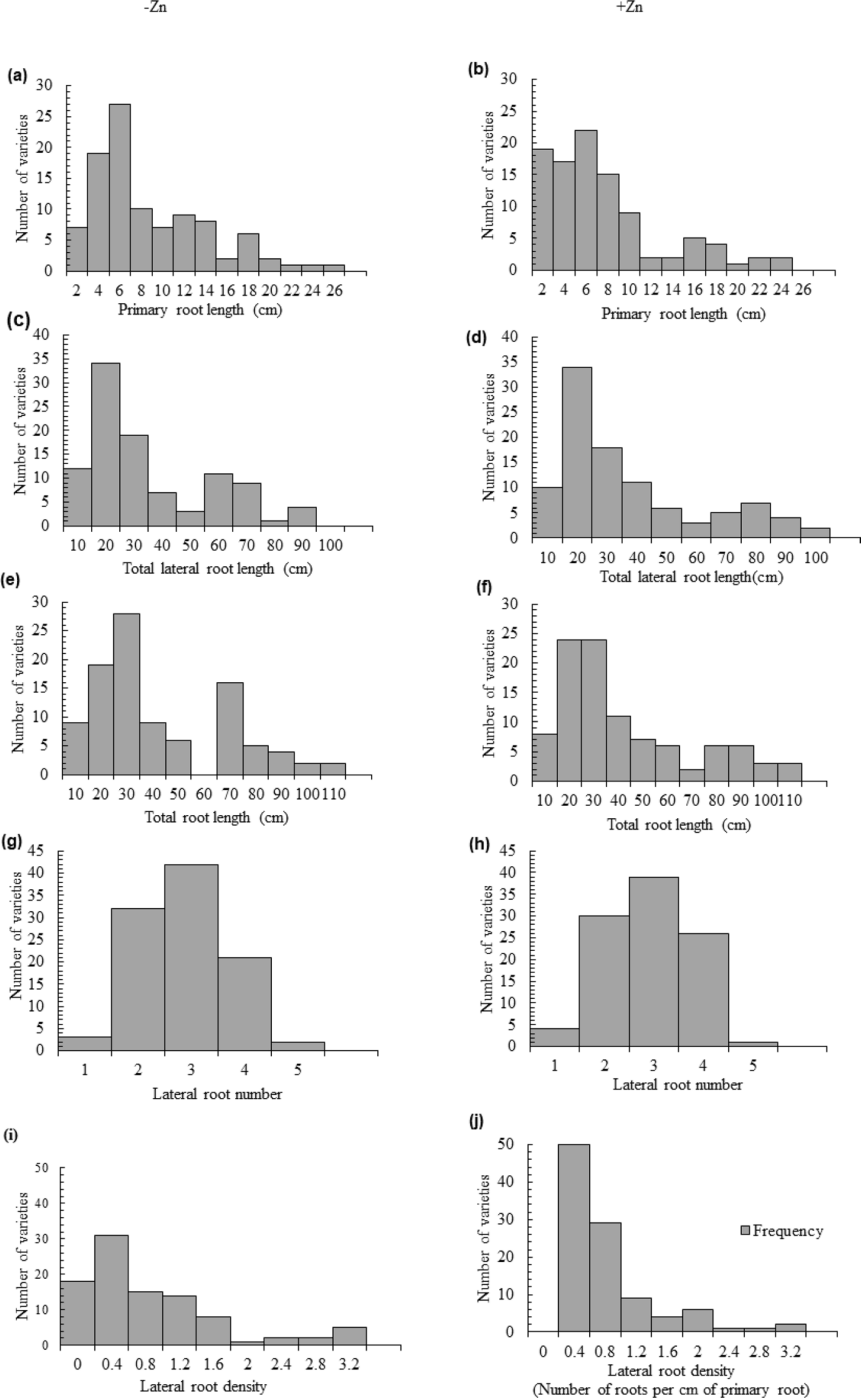
Frequency distribution histogram representing root system architecture of hundred varieties of wheat: Displaying counts of categorical quantitative single variable. Where columns correspond to bins that together span the range of the data, height of each bar corresponding to the number of data points falling into each bin.

The frequency distribution for primary root length (PRL), lateral root length (LRL), total root length (TRL), and lateral root density (LRD) was right skewed under Zn-sufficient and deficient conditions. The maximum number of varieties for PRL lies in the 4–6 cm range under both conditions. The largest PRL was observed in Zincol-16 (24.83 cm), whereas the smallest PRL was detected in Shafaq-2006 (1.00 cm) in Zn deficient conditions (Fig. 2a). Whereas in Zn sufficient condition, the largest PRL was observed in NARC-2011 (23.46 cm) and the smallest PRL was detected in Shafaq-2006 (0.58 cm) (Fig. 2b).

TLRL ranging from 10 to 30 cm was recorded in the maximum number of varieties in both Zn sufficient and deficient treatments. The largest TLRL was detected in Zincol-16 (89.61 cm), whereas the minimum TLRL was observed in Faisalabad-08 (4.72 cm) under Zn deficient conditions (Fig. 2c). In Zn-sufficient conditions, NIA Sunder shows the highest TLRL (95.79 cm), and the minimum TLRL was seen in AARI-2010 (4.11 cm) (Fig. 2d). The largest TRL was observed in Janbaz (107.89 cm), the smallest TRL was observed in Shafaq-2006 (5.78 cm) under Zn deficient conditions (Fig. 2e). Under Zn sufficient conditions, the largest TRL was observed in NIA Sunder at 109.93 cm, and the smallest TRL was observed in AARI-2010 at 5.25 cm (Fig. 2f).

Frequency distribution representation for LRN was normal distribution in the histogram (Fig. 2g, h). Maximum varieties produce three lateral roots. Maximum LRN was observed by Chakwal-97 and Chakwal-50 (5), and minimum LRN was exhibited by BARS-2009, Aas-2009, and Faisalabad-08 (1) in Zn-deficient conditions (Fig. 2g). Under Zn sufficient conditions, higher LRN was seen in Sehar-06 (5), Tatara (4), and Atta Habib (4), and less LRN was observed in Abadgar (1), Auqab 2000 (1), NN-Gundum I (1) (Fig. 2h).

The maximum number of wheat varieties was in the range of 0.2–0.4 cm^−1^ LRD. Maximum LRD was seen in Auqab-2000 (3.12 cm^−1^) and minimum LRD was recorded in Pakhtunkhwa-15 (0.08) under Zn deficient conditions (Fig. 2i). In a Zn sufficient environment, maximum LRD was seen in Lasani-08, Suleman-96, and Abadgar (2 cm^−1^) and minimum LRD (0.1 cm^−1^) was observed in Pakhtunkhawa-15, NARC-2011, and Ihsan-16 (Fig. 2j).

### Correlation of attributes of root system architecture and grain Zn content

**Figure 3:**
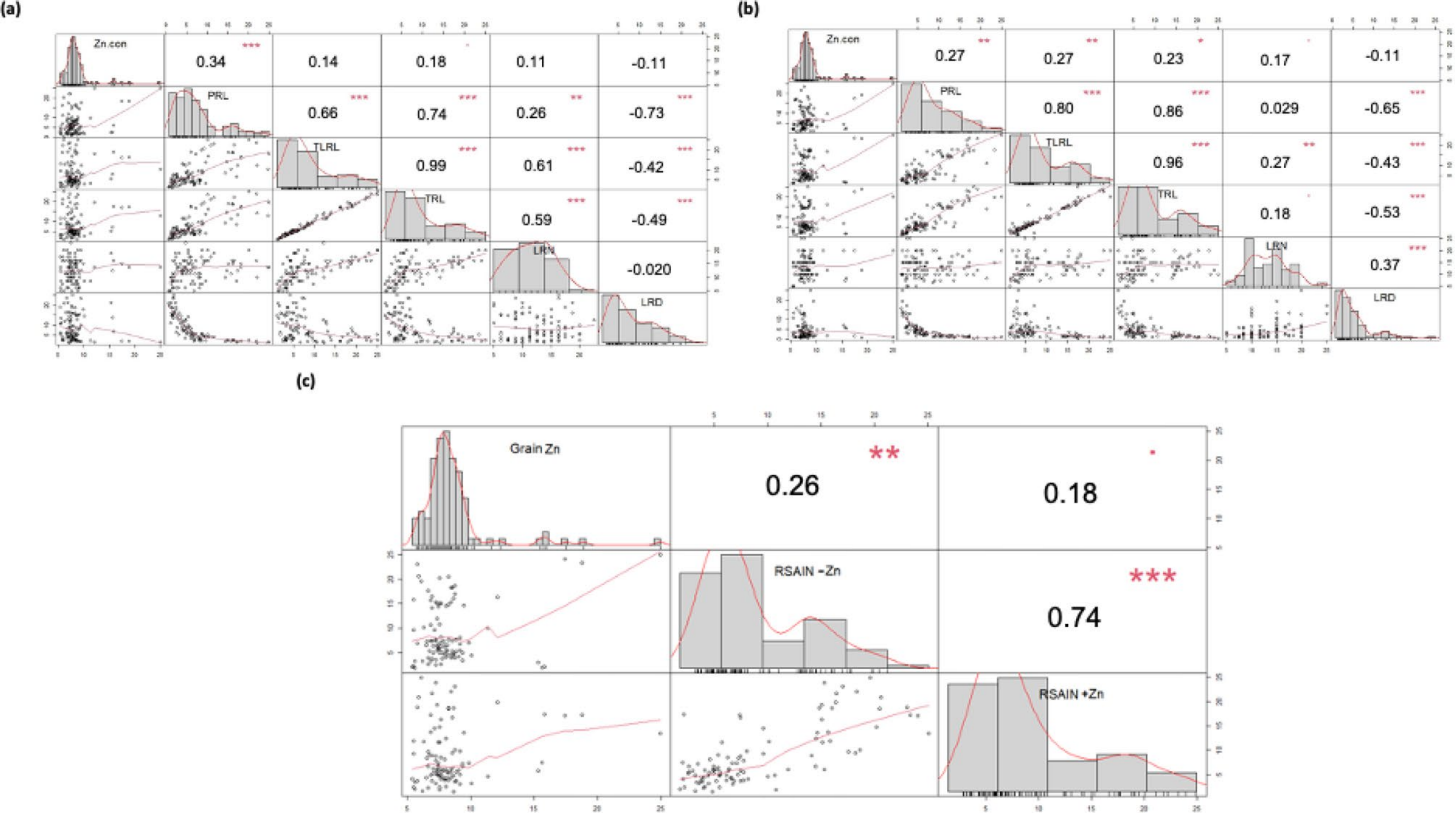
Pearson correlation matrix for the relationship of root system architectural attributes with grain Zn content in hundred wheat varieties. (**a**) RSA versus Grain Zn under Zn sufficient conditions, (**b**) RSA versus Grain Zn under Zn deficient conditions, (**c**) Cumulative RSA versus Zn sufficient and deficient conditions with Zn concentration. Diagonal representative features trend, upper section of diagonal representing coefficient of R^2^, Lower section of diagonal specify scatter plot with tendency line. Where Zn: Zinc concentration, TRL: Total Root Length, PRL; Primary Root Length, LRN: Lateral Root Number, TLRL: Total Lateral Root Length, LRD: Lateral Root Density, RSAIN -Zn: Root system architecture index with no Zn, RSAIN + Zn: Root system architecture index with Zn, Grain Zn: grain Zinc concentration.

Comparison of grain Zn content with root system architecture in Zn sufficient condition showed positive correlation with primary root length (R^2^ = 0.34), total lateral root length (R^2^ = 0.14), total root length (R^2^ = 0.18), lateral root density (R^2^ = 0.11) and negative correlation with lateral root number (R^2^ = − 0.11) (Fig. 3a). Whereas comparison of grain Zn content with RSA attributes in Zn deficient conditions showed positive correlation with primary root length (R^2^ = 0.27), total lateral root length (R^2^ = 0.27), total root length (R^2^ = 0.23), lateral root density (R^2^ = 0.17) and negative correlation with lateral root number (R^2^ = − 0.11) (Fig. 3b). Comparison of grain Zn content have positive correlation with cumulative root system attributes under Zn deficient conditions (R^2^ = 0.26) and Zn sufficient conditions (R^2^ = 0.18). Comparison of cumulative root system under Zn deficient and sufficient conditions have strong positive correlation (R^2^ = 0.74) (Fig. 3c).

### Screening of varieties with reference to root system architecture vs. grain Zn content

**Figure 4:**
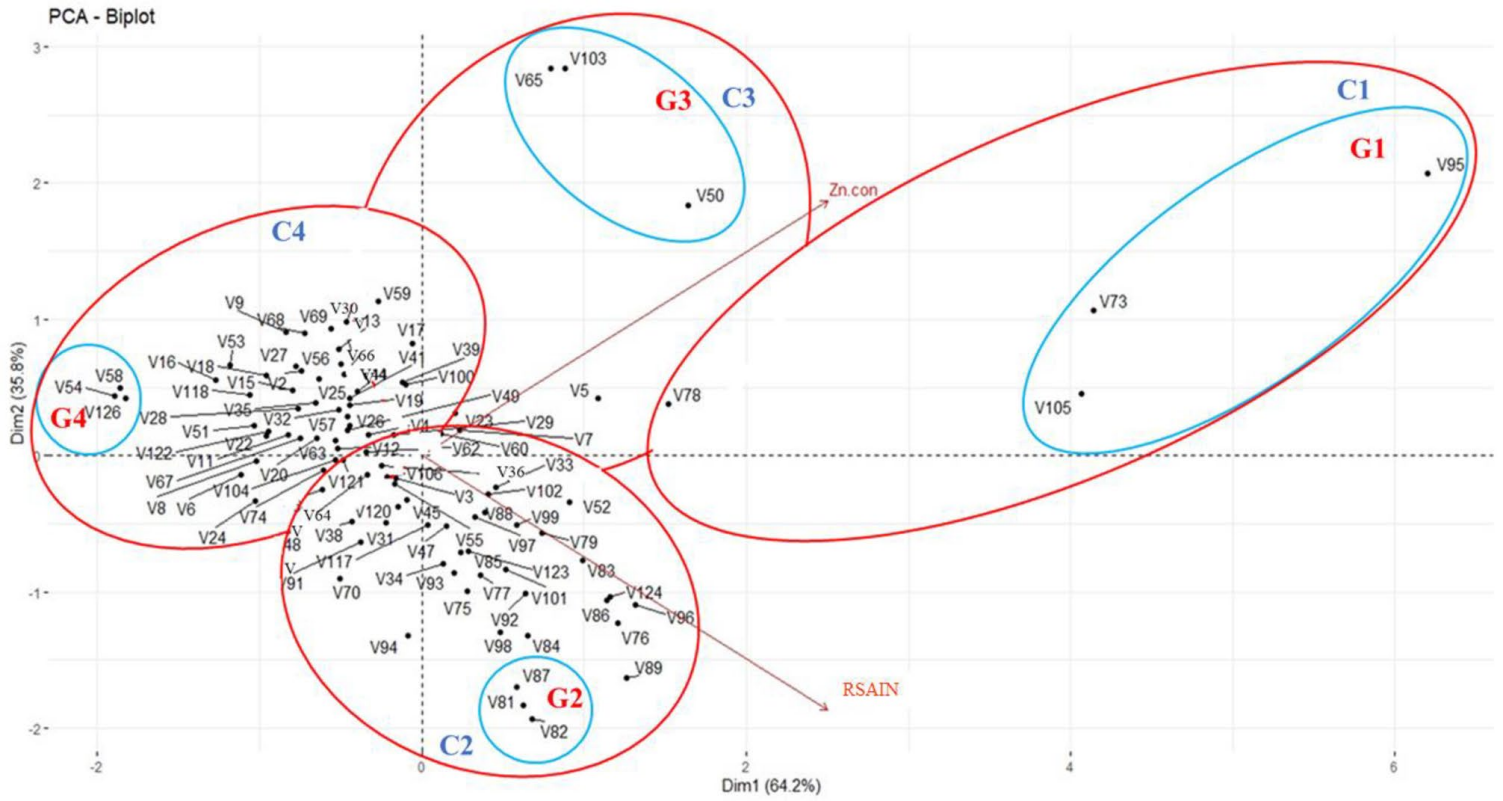
Principal component analysis between RSA and grain Zn content in hundred wheat varieties. C1: cluster 1; higher grain Zn content versus vigorous root system architecture, C2: cluster II; lower grain Zn content versus vigorous root system architecture, C3: cluster III; higher grain Zn content versus weaker root system architecture, C4: cluster IV; lower grain Zn content vs weak root system architecture. Screened twelve wheat varieties: G1; vigorous RSA versus higher grain Zn content, G2; vigorous RSA versus lower grain Zn content, G3; weaker RSA *vs* higher grain Zn content, G4; weaker RSA versus lower grain Zn content. weaker RSA *vs* higher grain Zn content.

The data was analyzed by principal component analysis to categorize the wheat varieties based on their Zn contents and RSA behavior. Four groups with three varieties in each were clustered together (Fig. 4). However, 4% of varieties showed higher grain Zn content and vigorous RSA amongst these varieties; Fatehjang-16 (V105), Punjab-11 (V73) and Zincol-16 (V95) were more efficient (G1). While 19% of varieties exhibited lower grain Zn with vigorous RSA, Pirsabak-13 (V82), NIA-Sunder (V5), and Shakar-13 (V81) were found on top (G2). Similarly, 8% of varieties have higher grain Zn content and weaker RSA, among which TD-1 (V65), Jauhar-16 (V103), Mairaj-08 (V50) were present (G3). Furthermore, 69% of varieties have low grain Zn concentration and weaker RSA, among which BARS-09 (V58), Aas-09 (V54), Fsd-08 (V126) exhibit the weakest attributes (G4) (Fig. 4).

### Study II Evaluation for Zn uptake and translocation to shoot and grains in wheat varieties with varying root system architecture

**Figure 5:**
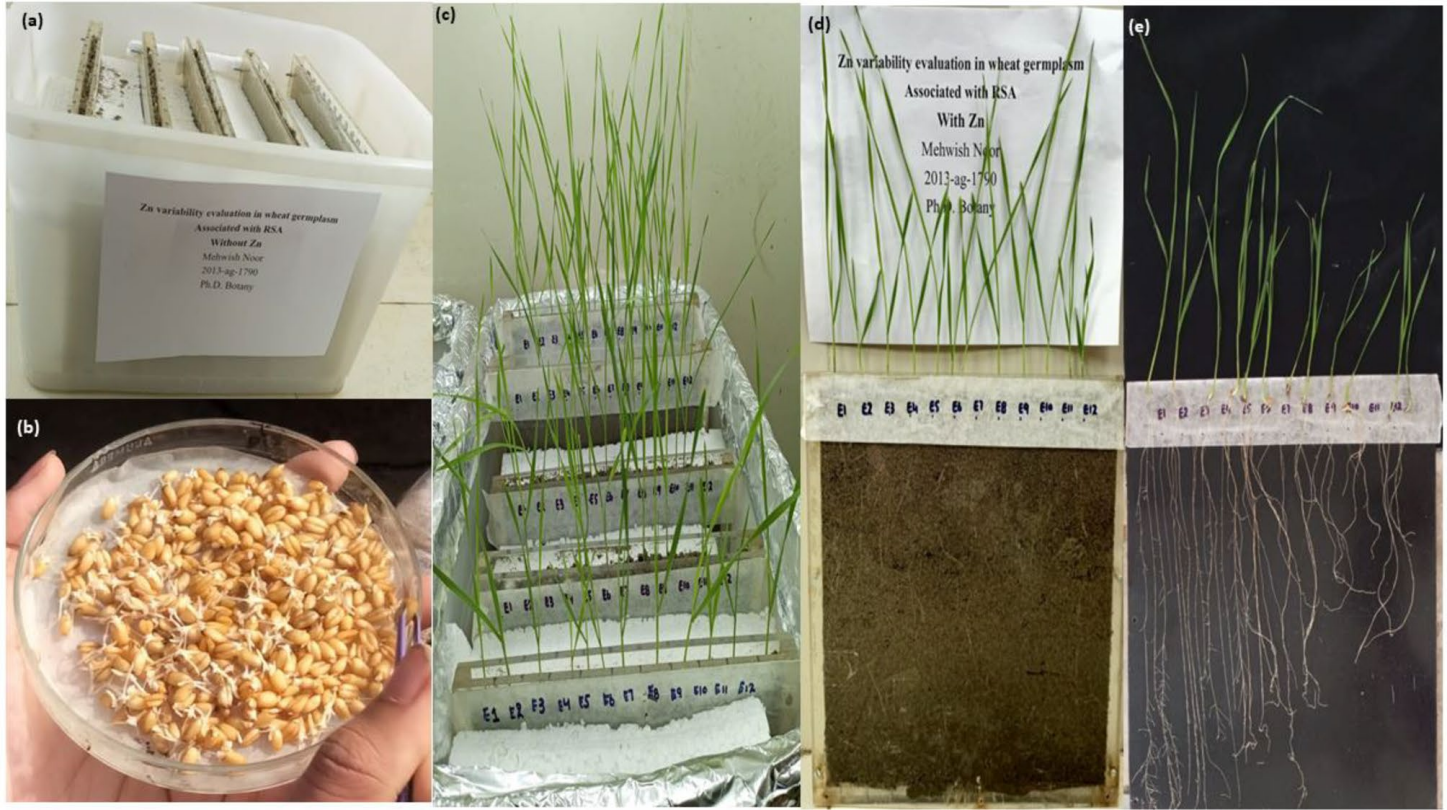
(**a**) Rhizobox arrangement for the study of 100 wheat varieties, (**b**) germinated seed transferred to rhizobox, (**c**) 12 days seedlings at harvesting stage grown in the rhizobox, (**d**) visual observation of roots in the sand filled rhizobox (with transparent plastic glass sheet), (**e**) washed roots of all the varieties taken out from the rhizobox for imaging.

From the results of Study I, twelve wheat varieties were selected after evaluating the data using PCA. Three varieties of wheat from each of the following categories were selected from the rhizobox experiment for the pot experiment: A pictorial representation of the RSA of selected varieties has been presented in Fig. 5, and the mean values of the RSA attributes are given in the supplementary file (Table S1). Screened wheat varieties selected from rhizobox experiment are G1: Higher Zn content of grains and vigorous RSA (Punjab-11, Fatehjang-16, Zincol-16), G2: Lower Zn content of grains and vigorous RSA (Pirsabak-13, NIA-sunder, Shakar-13), G3: Higher Zn content of grains and weaker RSA (TD-01, Jauhar-16, Mairaj-08) and G4: Lower Zn content of grains and weaker RSA (BARS-09, Aas-09, Fsd-08).

### Yield parameters

**Figure 6:**
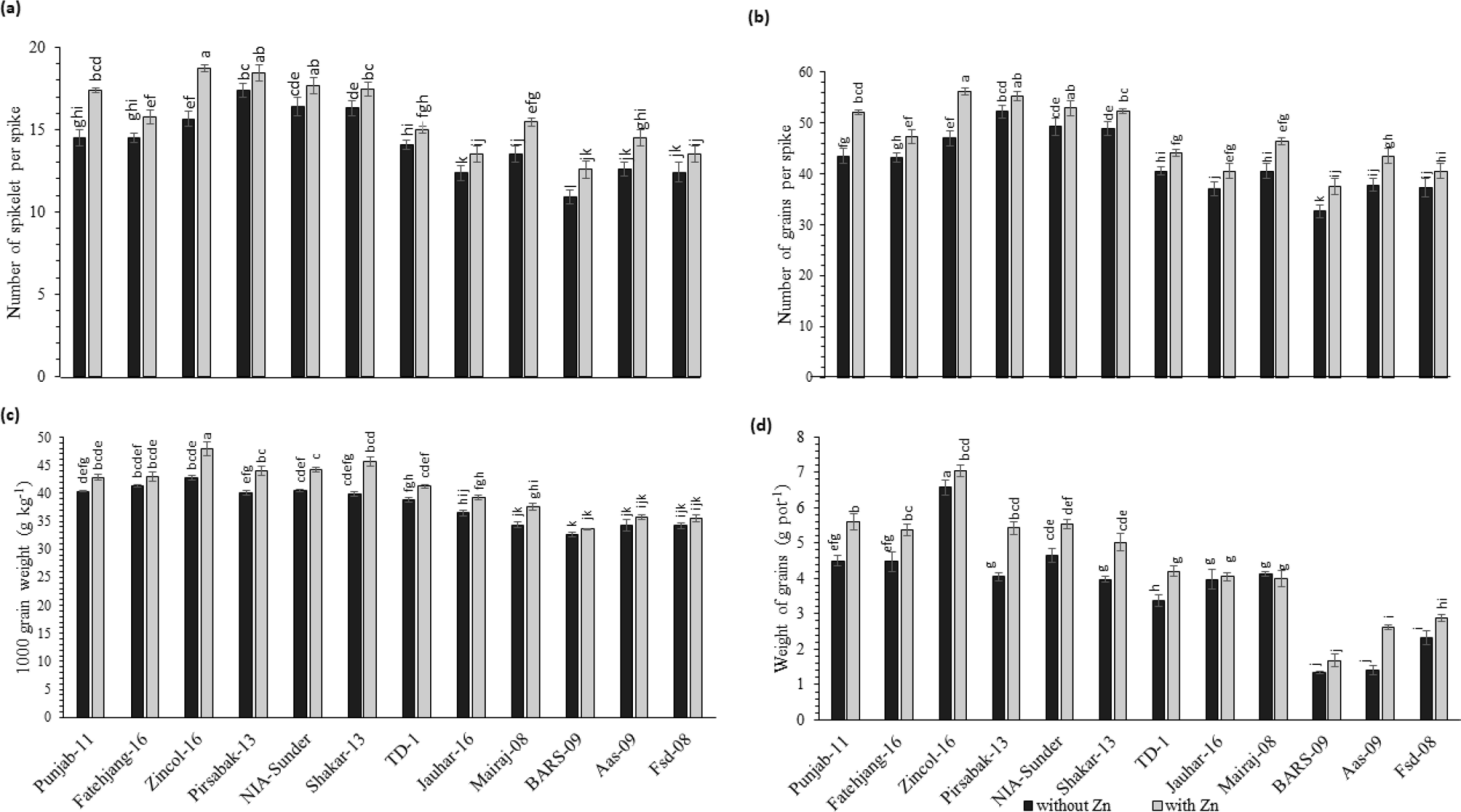
Zn application effect on yield traits of various wheat varieties in pot study. Treatment: Without Zn application and with Zn application at proportion of 6 kg ha^−1^. Where (**a**) Number of spikelets per spike, (**b**) Number of grains per spike, (**c**) weight of grains (g pot^−1^), (**d**) 1000 grain weight (g). Value in column representing the mean of four replications, grey column displaying with Zn application and black column with Zn application, while bars display the standard error, column sharing letter (s) are significantly different at *p* < 0.05. *LSD value*: Number of spikelets per spike:1.334, Number of grains per spike: 2.698, weight of grains: 0.1587, 1000 grain weight: 1.545.

Yields of selected varieties were monitored in pot experiments under Zn variation. Spikelet numbers ranged from 10 to 20 among different varieties. The maximum spikelet number was found in Pirsabak-13 (17) and the minimum in BARS-09 (11) in cases of Zn deficient conditions. Whereas, in Zn sufficient condition, maximum numbers were found in Zincol-16 (19), and minimums in BARS-09 (13) (Fig. 6a). Statistical analysis revealed that the number of grains per pot increased under Zn sufficient conditions as compared to Zn deficient situations (Fig. 6b). The maximum number of grains was 56 in Zincol-16, and the minimum was 38 in BARS-09 under Zn availability. Under no Zn application, maximum grains were found in Pirsabak-13 (52) and minimums in BARS-09 (33). It was also observed from the results that 1000 grains weight improved under Zn as compared with no application of Zn. The maximum 1000 grain weight was recorded in Zincol-16 (48 g) and the minimum was recorded in BARS-09 (32.6 g) (Fig. 6d).

### Zn concentration in different parts of wheat

**Figure 7:**
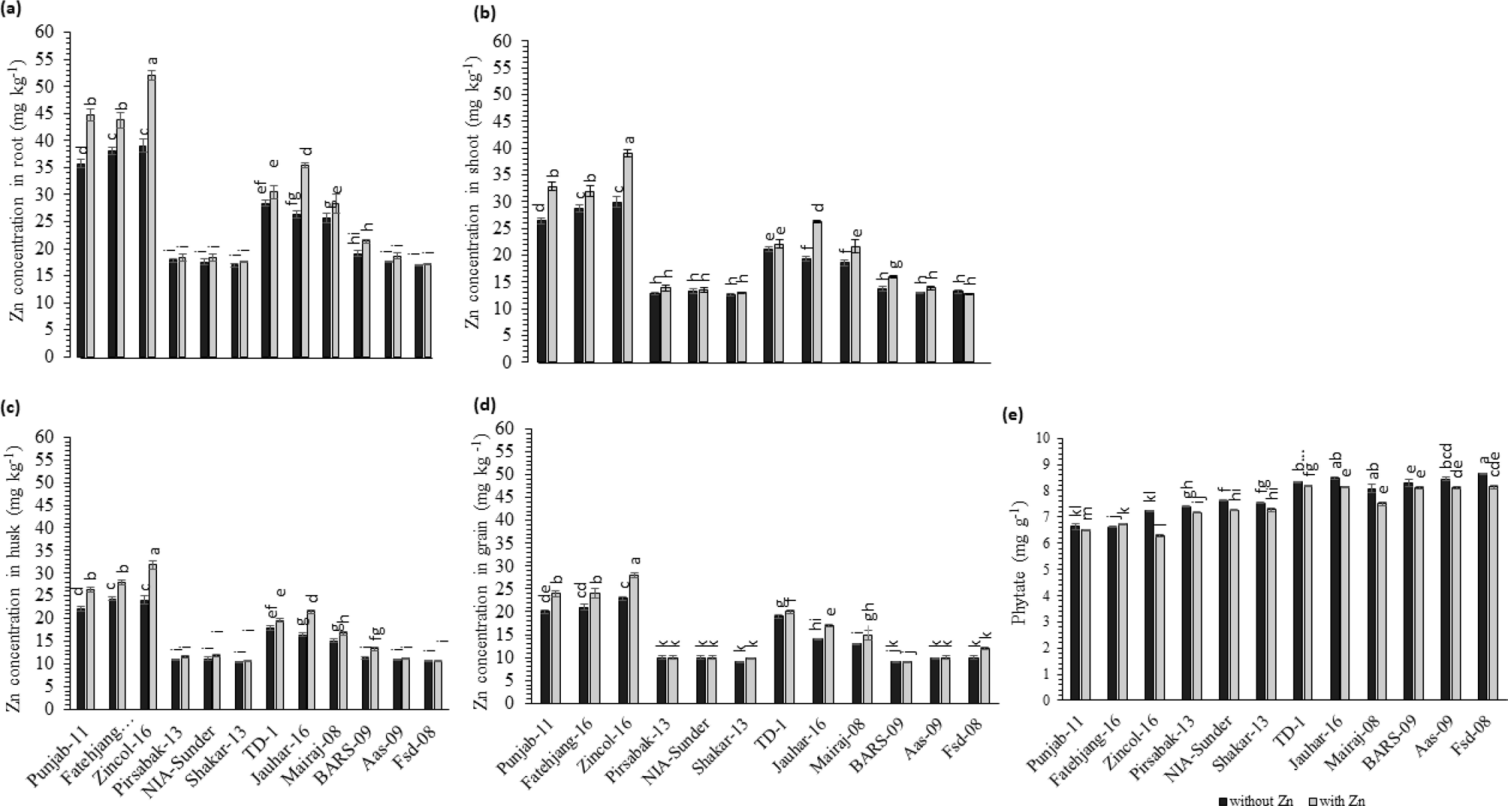
Zn application effect on Zn uptake and translocation in different wheat varieties in green house environment. Treatment: Without application of Zn and with application of Zn at rate of 11 mg ZnSO_4_ kg^−1^ where (**a**) Zn concentration in root, (**b**) Zn concentration in shoot, (**c**) Zn concentration in husk, (**d**) Zn concentration in grain, (**e**) Phytate concentration. Value in column represents the mean of three replication, black column representing without Zn application and grey column with Zn application, while bars display the standard error, column sharing letter (s) are significantly different at *p* < 0.05. *LSD value: Root Zn* = *2.202, shoot Zn* = *1.649, husk Zn* = *1.365, grain Zn* = *1.177, Phytate* = *0.185.*

Zn concentration was examined in roots, shoots, husks, and grains to evaluate the translocation of Zn. Root and shoot analysis for Zn concentration was done for the assessment of Zn translocation from root to shoot and husk to grain. The maximum Zn concentration was found in Zincol-16 in root (52.10 mg kg^−1^; Fig. 7a), shoot (39.03 mg kg^−1^; Fig. 7b), husk (31.84 mg kg^−1^; Fig. 7c) and grain (28.07 mg kg^−1^; Fig. 7d). Whereas the minimum Zn concentration of root, shoot, husk, and grain was found in Shakar-13 (16.91 mg kg^−1^; Fig. 7a, 12.96 mg kg^−1^; Fig. 7b, 10.36 mg kg^−1^; Fig. 7c, and 9.35 mg kg^−1^; Fig. 7d) respectively. Phytate contents increased under Zn available conditions in all varieties (Fig. 7e). The highest phytate concentration (8.65 mg kg^−1^) was found in Fsd-08 and lowest in Fatehjang-16 (6.61 mg kg^−1^) under Zn deficient conditions. Whereas in Zn sufficient condition, maximum phytate concentration was observed in TD-01 (8.20 mg kg^−1^) and minimum in Zincol-16 (6.74 mg kg^−1^).

### Zn uptake and translocation in wheat

**Figure 8:**
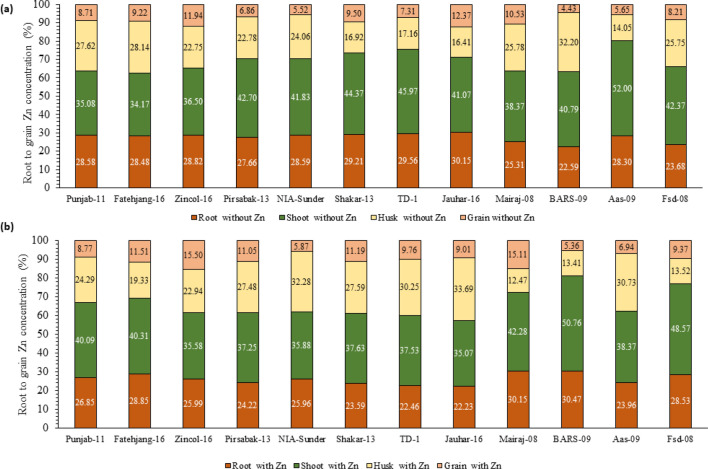
Zinc acquisition and translocation by different varieties of wheat grown in case of soil application of Zn in pot study. Zn application at rate 11 mg kg^−1^ soil, Zn acquisition and translocation showed on grouped stacked column chart representing visual Zn content comparison from root to shoot. Data values on X axis showing total Zn uptake by plant. (**a**) Uptake and translocation of Zn by various wheat varieties grown in conditions without soil application of Zn in pot experiment. (**b**) Uptake and translocation of Zn by various wheat varieties grown in conditions with soil application of Zn in pot experiment.

Proportional analysis of Zn absorption and translocation from root to grain revealed variability in Zn acquisition in selected wheat varieties (Fig. 8). Efficient wheat varieties can uptake more Zn by roots and translocate more Zn concentration to the direction of distant sinks, i.e., shoot, husk, and grain. In Zn-sufficient conditions, the highest Zn was absorbed and translocated by Zincol-16 from root (25.99%) to shoot (35.58%), shoot to husk (21.94%), and husk to grain (15.50%), whereas at the same time the lowest Zn was translocated in BARS-09 (root: 30.47%; shoot: 50.76%; husk: 13.41%; grain: 5.36%). In Zn-deficient conditions, the same trend was observed as the highest Zn was absorbed and translocated in Zincol-16 from root (28.82%) to shoot (36.50%), shoot to husk (22.75%), and husk to grain (11.94%), whereas the lowest minimum Zn was translocated in BARS-09 as 40.79% of Zn was in shoot and 32.2% in husk, while only 4.43% Zn was present in grain.

### Zn localization

**Figure 9:**
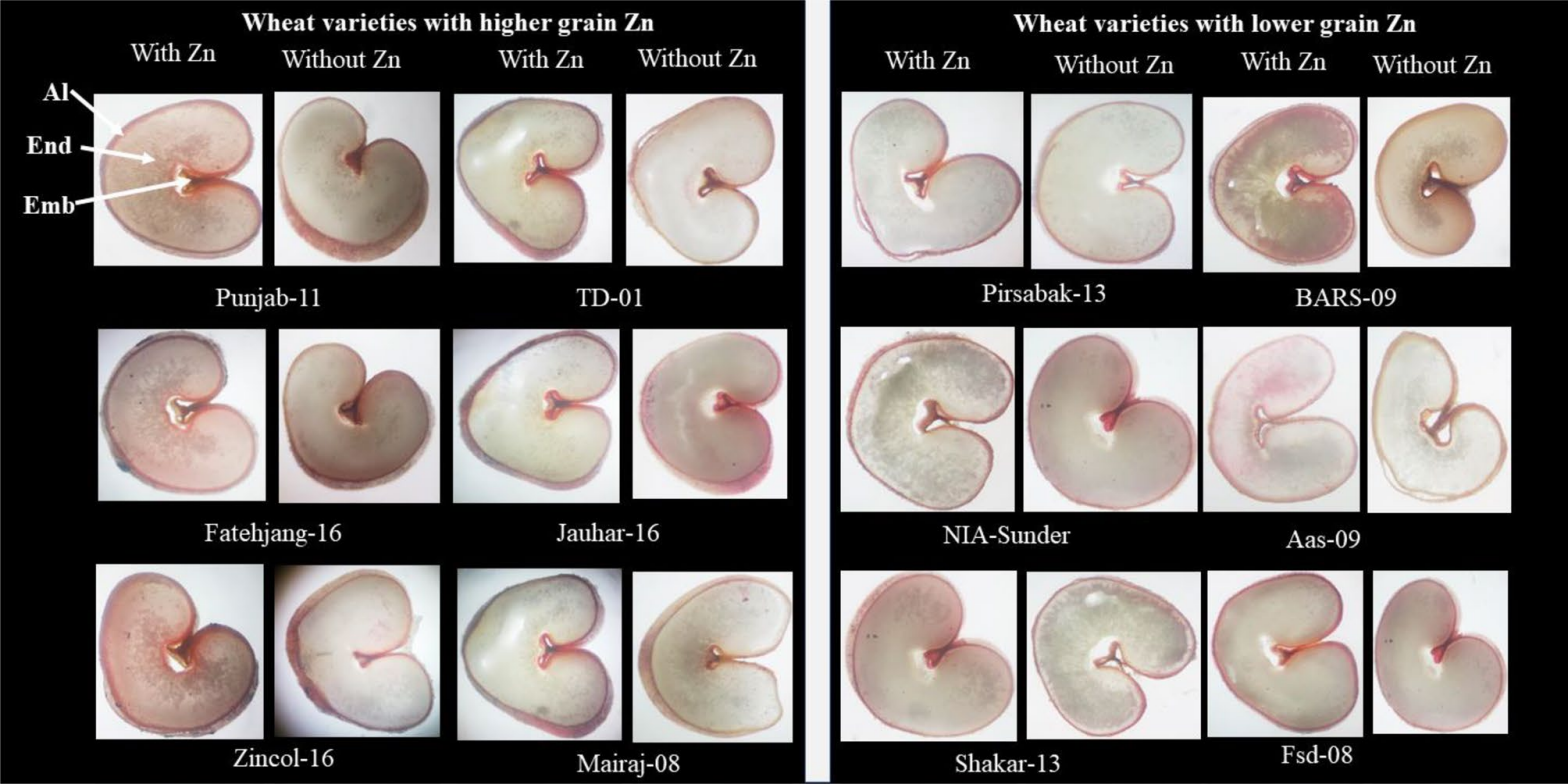
Zn localization in wheat grain of twelve varieties as affected by Zn application by Euromex stereoblue (110–240 V/50–60 Hz). Red color due to diphenyl thiocarbazone (DTZ) stain is specific for Zn in wheat seeds. (*Al* aleurone layer, *End* endospermic region, *Emb* embryonic region).

Microscopic observations for localized Zn with stains revealed that differentially localized Zn could be observed in the aleurone layer, crease scutellum, and endospermic regions of wheat kernels under Zn treatments. Grains with higher concentrations of Zn looked darker in color as compared with the grains with lower concentrations of Zn. Zincol-16 showed a darker stain in the aleurone layer than other varieties, whereas, BARS-09 aleurone layer stained lighter (Fig. 9).

## Discussion

Roots, being the hidden half of plants, are considered a vital part of developing strategies related to nutrient uptake by various crops, including staples such as wheat. A better understanding of root genetics can be helpful to identify wheat varieties with vigorous RSA that are effective for micronutrient uptake, such as Zn^19^. Root plasticity is helpful in a normal or low-input environment where the accessibility of soil nutrients is limited to plants^20^. Genetic variability as well as environmental conditions contribute to the development of diverse RSA in heterogeneous rhizospheres^21^, and this relationship limits crop yields and quality^22^. Phenotypic analysis of root seedlings can evaluate the rhizospheric resources acquired by roots. However, grain-stored nutrients are also a primary and significant source of nutrition during early growth stages^23^.

Zinc-efficient wheat varieties exhibited vigorous root volumes as compared to Zn-inefficient wheat varieties under inadequate supply of Zn, helping plants acquire immobile Zn resources in the soil (Guo et al. 2020; Fig. 1)^24^. It is the thrust for Zn produced in plants due to Zn deficiency in the rhizosphere, as studied by Nanda and Wissuwa, (2016). Since fluctuations in Zn levels regulate the cellular expression and distribution of Zn transporters^25,26^. Hence, plants modify their root growth and development to improve their capacity to take up nutrients under stressful conditions^27^. Furthermore, Norman et al. (2017) found a correlation between grain nutrient reserves and diverse RSA^28^.

The genotypic variation in primary root length, total root length, total lateral root length, lateral root number, lateral root density, and grain Zn concentration observed in 100 wheat varieties (Figs. 1 and 2) provides a base to identify the required RSA traits. Diversification of RSA among different varieties is responsible for seedling emergence and adapting complex rhizospheres^29^. Zinc-efficient varieties utilize and mine Zn efficiently by increasing acquisition of Zn in the rhizosphere through modification of root system architecture^30^. There is variation in the ability for acquisition and translocation of nutrients among different varieties because the root phenes are specific traits for an individual variety that direct the wheat plant’s temporal and spatial distribution of roots for mining of the nutrients^31^. Wheat varieties with vigorous RSA had high grain Zn concentrations (Fig. 7), because longer and deeper RSA can acquire more available Zn from the soil, and translocate it to grain, improving growth, yield, and grain Zn concentration^28^. Since Zn is an immobile micronutrient, topsoil growth of roots helps plants to capture immobile resources in sufficient amounts^10^, and the number and length of lateral roots may play a significant role (Fig. 2). Under Zn sufficient conditions PRL was the major attribute contributing to grain Zn concentration, while under Zn deficient condition, lateral root length was the major contributor to grain Zn (Fig. 3). Varieties with vigorous root system led to higher grain yield, though the grain Zn concentration were variable, while all varieties with weaker root system had lower yield as well as grain Zn concentration (Figs. 6 and 7), owing to genotypic variation, nutrients availability, and the biochemistry of the rhizosphere^32^. More cations (H^+^ ions) are absorbed and OH^−^ are excreted by roots, leading to a decrease in the acidification of the rhizosphere. Therefore, soil-to-root cation–anion balance is disturbed, and consequently, low uptake of minerals takes place under vigorous RSA^33^.

Wheat varieties responded positively to Zn fertilization; however, again, variable responses were observed among different varieties. Zincol-2016, a Zn biofortified wheat variety, was selected in the category of high Zn supported by vigorous RSA and performed more efficiently than other varieties. Improvement was observed in grain Zn concentration (29.86%) as well as in yield (21.02%) compared with control after Zn fertilization (Figs. 6 and 8). Xiong et al. (2006) examined both traditional and new Chinese wheat cultivars and found a positive relationship between grain yield and RSA^34^. Previous studies also reported that application of Zn as a basal dose enhanced grain Zn concentration and quality attributes of wheat grain^5,15^. Niyigaba et al. (2019) also observed that fertilization with Zn enhanced wheat grain yield, which might be due to Zn’s involvement in enzyme activation^35^. So, it is deduced from all these studies that soil Zn fertilization not only enhanced grain Zn concentration but also improved yield parameters of wheat^36^. In fact, the role of the RSA in nutrient uptake efficiency and yield in different circumstances cannot be denied^37^, even under Zn-fertilized conditions. Other factors responsible for variable Zn concentration in grain despite the fertilization of an equivalent Zn dose may be due to phytate content in grain because an antagonistic relationship exists between grain Zn and phytate availability^38^. The lowest grain Zn-containing variety (BARS-09) produced 28.98% more phytate content than the highest grain Zn variety (Zincol-16) (Fig. 8). However, Zn reduces phosphorus uptake; therefore, decreased phytate content in varieties with high Zn content can be attributed to low P uptake^8^. Studies involving regulatory mechanisms show that Zn-sufficient plants appear to build capacity to downregulate expression of genes encoding high-affinity P transporters (PT1 and PT2) in roots^39^.

Comparatively more Zn was localized in the aleurone layer and embryonic region than endosperm, as visualized by simple staining techniques (Fig. 9), also previously reported by Kiran et al. (2021), whereas endosperm, the vital part of wheat grain consumed by humans through bread flour, contains very low levels of Zn due to the removal of the aleurone layer and embryo during the milling process^5,40^. Appropriate strategies need to be adopted for the improvement of Zn concentration considering the genotypic variation of endospermic Zn content through agronomic biofortification.

## Conclusion

The RSA not only affects growth, yield, and biological attributes but also influences Zn acquisition, leading to its translocation towards shoot and grain. Based on the information achieved, Zn-efficient wheat varieties with novel Zn-specific RSA can be sorted out for further use in breeding. Since Zn biofortification of wheat grain is the main target in many developing countries, considering RSA attributes to develop Zn-efficient varieties should be included in breeding programs. Furthermore, it can also be mentioned here that agronomic Zn biofortification through Zn fertilization of plant roots can lead to a significant increase in grain Zn concentration and should be included as a necessary input along with other fertilizers under Zn-deficient soil conditions. However, to enhance the Zn content in endosperm, further genetic improvement may be considered to achieve the targets for sufficient Zn supply to the population of developing countries through staples.

## Material and methods

This study was planned to determine variation in root system architecture (RSA) attributes and their association with genotypic variability in grain zinc (Zn) content. One hundred varieties of wheat were obtained from the National Agriculture Research Center (NARC) in Islamabad, Pakistan, for conducting two experiments. Parentage information for these varieties is given in the supplementary file (Table S1).

In Study I, Grain Zn was determined by an atomic absorption spectrophotometer (Hitachi, Japan) in these available wheat varieties by following the protocol given by Uddin et al.^41^. Root system phenotyping was optimized for these varieties in sand-filled rhizoboxes (Fig. 5) Hoagland nutrient solution with and without Zn was applied, to check the relationship between RSA and Zn in wheat germplasm. Seeds were germinated in petri plates, and twelve varieties of wheat were transplanted to each irrigated rhizobox with four replicates (Fig. 5a). Seedling growth was monitored in the growth room of the Department of Botany, University of Agriculture Faisalabad during 2018–2019. Seedlings were harvested after 12 days (Fig. 5).

Images of roots and shoots were taken, immediately after harvesting, by using camera (Nikon Coa-PTX L840). The images were subjected to analysis for RSA attributes by using Image J based software SmartRoot^42^. Data on root system architecture attributes, i.e., Primary root length, total lateral root length, total root length, lateral root number, and lateral root density, was recorded.

Study II was conducted for the evaluation of Zn uptake and translocation from root to shoot to grain in wheat varieties with varying root system architecture. This study was a soil-filled pot experiment with a completely randomized design (CRD) carried out in the green house of the Department of Botany, University of Agriculture Faisalabad, Pakistan during 2019–2020. For this pot experiment, three varieties of wheat from each of the following categories were selected from the rhizobox experiment: 1) higher grain Zn and vigorous RSA; 2) lower grain Zn and vigorous RSA; 3) higher grain Zn and weaker RSA; 4) lower grain Zn and weaker RSA.

Soil (60%) and sand (40%) were sieved, mixed, and filled in pots (8 kg). Fertilizers to fulfill the nitrogen (N), phosphorus (P), and potassium (K) requirements were applied in the form of urea (46% N) 163 mg urea kg^−1^ soil, single superphosphate (18% P_2_O_5_) 277 mg SSP kg^−1^ soil and sulfate of potash (50% K_2_O) 60 mg SOP kg^−1^ soil at a rate of 150:100:60 kg ha^−1^ respectively. Phosphorous, potassium, and a quarter dose of N were applied as a basal dose. A second N dose at the time of tillering and a third dose at the time of heading stage were applied, respectively. There were two treatments, i.e., Zn deficient (without Zn fertilizer) and Zn sufficient soil conditions, where ZnSO_4_ (11 mg kg^−1^ soil) was applied as the basal dose in four replicates. Three plants per pot were maintained. Morphological, physiological, and yield parameters were recoded. Standard crop management practices were followed. At maturity, all plants from every pot were harvested, and samples were processed further for biochemical analysis and Zn localization in grains.

### Biochemical analysis

Wheat grains were oven-dried for 2 h at 60–70 °C. The powder of the samples (0.25 g) was mixed in 2.5 mL of a 2:1nitric acid and perchloric acid mixture in a digestion flask. Samples were kept overnight. These samples were heated at 180 °C until white fumes formed. Then samples were allowed to cool down and reach a volume of 50 mL by adding deionized water. These samples were preserved after filtration for further analysis. A blank solution was prepared by following the same procedure. Zinc analysis of the samples was estimated by an atomic absorption spectrophotometer (analytic jena, AA 350) from the Hitech lab of the Depalpur campus, University of Agriculture Faisalabad (UAF), following the atomic absorption method^43^. Phytate content was determined using a UV visible spectrophotometer (Aquarius CE 7400, 7000 Series) from the Environmental Biogeochemistry Lab UAF.

Dithizone reagent was used for zinc staining of seed in this study as described by Ozturk et al. (2006). Grain samples were soaked in water for 2 h. Cross sections of grains were kept in petri plates containing 500 mgL^−1^ Diphenyl thiocarbazone (DTZ) at normal temperature for 30 min. Stained sections were examined using a high-resolution digital camera reflectance light microscope (Stereo Blue Euromex model). Pinkish color appears when DTZ reacts with Zn and reveals the location of Zn in the embryonic part, aleurone layer, and other parts of seeds^44^.

### Statistical analyses

Statistical analysis was carried out using R Studio 4.0.3^45^. Principal component analysis (PCA) was applied to data from Study I for the selection of four categories. An analysis of variance was applied to the whole dataset from the pot experiment under the CRD design. The comparison of treatments was carried out by means of the least significant difference (LSD) test by considering a probability level of 0.05^46^.

### Research involving plants

Wheat germplasm used in this research was obtained from the National Agriculture Research Center (NARC) in Islamabad, Pakistan and comply with relevant institutional, national, and international guidelines and legislation.

### Supplementary Information

Supplementary Information.

## Data Availability

All data generated or analyzed during this study and its supplementary information files available from the corresponding author on reasonable request.
