# Supplementary material for: Root system architecture associated zinc variability in wheat (Triticum aestivum L.)
Source: Sci Rep. 2024 Jan 20;14:1781. doi: 10.1038/s41598-024-52338-3 (PMC10799890; doi:10.1038/s41598-024-52338-3)
Supplement: Supplementary file 1 — Supplementary Information. [file 41598_2024_52338_MOESM1_ESM.pdf]

Root System Architecture Associated Zinc Variability in Wheat (*Triticum aestivum* L.)

Mehwish Noor[1], Aysha Kiran[1*], Muhammad Shahbaz[1], Muhammad Sanaullah[2] and Abdul Wakeel[2*]

[1]Department of Botany, University of Agriculture, Faisalabad, 38040 Pakistan
[2] Institute of Soil and Environmental Sciences, University of Agriculture, Faisalabad, 38040 Pakistan

*Corresponding author's email: aysha.kiran@uaf.edu.pk, abdul.wakeel@uaf.edu.pk

Table. S1. Wheat varieties selected for the study of corelation of root system architecture with reference to grain Zn content.

Sr. No	Varieties	Label	Year of release	Parantage/Pedigree
1	Bakhtawar 92	V2	1994	JUP/BJYG//URES<br>CM 67458-4Y-1M-3Y-1M-5Y-0B
2	Chakwal 86	V3	1988	Fln/ACS//ANA<br>SWM4578-56M-3Y-3M-0Y-0PAK
3	Sind-81	V4	1983	Norento x Mexipak
4	Zarghoon-79	V5	1979	CC-Inia/Tobari-C.fon/BB<br>CM8237-G-1M-3Y-2M-4Y-0M
5	Faisalabad - 83	V6	1983	FURY//KAL/BB<br>CM 37138-48Y-1M-5Y-1M-4Y-5Y-0A
6	Faisalabad - 85	V7	1985	MAYA/'MON'S'//KVZ/TRM<br>CM 44083-N-3Y-1M-1Y-1M-1Y-0B
7	Watan	V8	1993	LU26/HD 2179
8	Kaghan - 93	V9	1993	TTR/JUN<br>CM59123-3M-1Y-2M-1Y-2M-2Y-0M-0PAK
9	Kohinoor -83	V11	1983	OREF1158/FDL/MFN/2*TIBA63/3/COC<br>CM37987-I-1Y-5M-OY-0PAK
10	LU - 26	V12	1976	BLS/KHUSHAL
11	Pasban - 90	V13	1991	INIA INIA66/A. DISTT//INIA66/3/GEN
12	Sarsabz	V15	1985	PI/FRND//MXP/3/PI/M20/70
13	Benazir - 13	V16	2013	CHEN/AEGILOPSSQARROSA (TAUS)//BCN/3/VEE#7/..<br>CMSS93B001854T-040Y-8Y-010M-010Y-010M-8Y-0M-5KBY
14	Tandojam – 83	V17	1983	BLUEJAYHS CM-5287-J-1J-2M-2Y-3M-0Y
15	MH - 97	V18	1998	ATTILAND/VG 9144//KAL/BB/3/YACO/4/VEE#5<br>CM. 85836-50Y-0M-0Y-3M-0Y
16	Kohistan - 97	V19	1997	V-1562//CHRC'S'/HORK/3/ KUFRA-1/4/CARP'S'/BJY'S'<br>PB. 24883B-1A-0A
17	Kohsar - 95	V20	1995	PSN/BOW<br>CM69560-1M-1Y-1M-2Y-0M-0(PAK)
18	Suleman - 96	V22	1996	F-6.74/BUN//SIS/3/VEE #7 (F6) CM86141-62M-0Y-0M-4Y-0M
19	Abadgar-93	V23	1996	CNO SIB/NO/3/C273//NP875/PI SIB/4/HD1981
20	Anmol-91	V24	1991	LIRA 'S'<br>CM-43903-H-4Y-1M-1Y-3M-3Y-0B
21	Bahawal pur -2000	V25	2000	AU/UP301//GLL/Sx/3/PEW „S"/4/MAI „S"/MAY A „S"//PEW"S"<br>CM.67245-C-2M-OY
22	Fakhr - e - Sarhad	V26	1997	PFAU"S"/SERI/BOW "S"<br>CM85295-010-TOPY-2M-0Y-0M-3Y-0M
23	Marvi-2000	V27	2004	CMH-77A917/PKV 1600//RL6010/6*SKA

24	Mehran - 89	V28	1989	VEERY 'S'<br>CM38027-F-15M-500Y-0M-87B-0Y
25	Soorab - 96 (Barley)	V29	1996	Soorab - 96 (Barley)
26	Tatara	V30	1996	JUP/ALD’S’//KLT’S’<br>CM79510-024Y-2M-05Y-01M-1Y-0B
27	Takbeer	V31	2000	Takbeer
28	Iqbal 2000	V32	2000	BURGUS/SORT 12-13//KAL/BB/3/PAK 81<br>PB 21912-11A-0A-0A-59A-0A-0
29	Auqab - 2000	V33	2000	CROW'S'/NAC//BOW'S'<br>PB 22138-3A-0A-0A-234A-0A
30	Chakwal - 97	V34	1997	BUC'S' / FCT'S'<br>CM84663-7M-0Y-0M-7Y-0M
31	Soghat - 90	V35	1990	PAVON MUTANT-3
32	Amin - 2008	V36	2008	PASTOR/OPATA<br>CM 110624-7M-020Y-010M-010SY-010M-0M-0Y
33	Zarlashata-99	V38	1999	Ures"s" / Bow<br>CM78108-1M-02Y-02M-22Y-3B-0Y
34	Wafaq -01	V39	2001	OPATA/RAYON//KAUZ<br>CMBW 90Y3180-0TOPM-3Y-010M-010M-010Y-1M-015Y-0Y
35	Durum-97	V41	1998	JO/AA//FG
36	Pirsabak - 2005	V44	2005	MUNIA/SHTO//AMSEL
37	Inqilab-91	V45	1991	WL 711/CROW "S"
38	Chakwal - 50	V47	2008	ATTILA/3/HUI/CARC//CHEN/CHTO/4/ATTILA<br>CMW90M48601-0T-TOPY-16M-1Y-010M-010Y-1M-015Y-0Y
39	Saussi	V48	2006	CHIL / ALD // PVN / Yecora-70
40	Lasani -08	V49	2008	LUAN/KOH97<br>PBP.29645-14A-18A-8A-4A-2A-0A
41	Meraj -08	V50	2008	SPARROW/INIA//V.7394/WI-711/3/BAU‟S‟<br>BR.2974-2B-1B-9B-0B
42	Fareed - 06	V51	2006	PTS/3/TOB/LFN//BB/4/BB/HD8325//ON/5/GV/ALD‟S‟//HPO‟S‟<br>BR-3385-3B-1B-OB
43	Pothowar-93	V52	1994	URES/BOW`S
44	Bathoor -08	V53	2008	PFAU/JUN//KAUZ<br>CM96818-1-0Y-0M-0B-2Y-2Y-0M
45	Aas - 2009	V54	2009	KHP/D31708//CM74A370/3/CIAN079/4/RL6043/*4NAC PBD<br>795-23A-1A-0A
46	NARC - 2009	V55	2009	INQALAB 91*2/TUKURU<br>CGSS99B00015F-099Y-099M-099Y-099M-29Y-0B-0ID
47	AARI-2010	V56	2010	SH-88/90A204//MH-97
48	NIFA-Barsat-10	V57	2010	FRET2<br>CGSS96Y00146T-099B-099Y-099B-16Y-0B-0SY
49	BARS-2009	V58	2009	PFAU/SERI//BOW<br>CM85295-101TOPY-2M-0Y-0M-3Y-0M-0SY
50	Daman -98	V59	1998	BOW”S”/3/CAR853/COC//VEE”S”,<br>CP02274-4C-0C-0Y-5M-ORES
51	Dera - 98	V60	1998	F12-71/COC//CNO79<br>CM76688-9Y-03M-02Y-2B-0Y
52	Zam-04	V62	2004	KAUZ* 2/OPATA//KAUZ<br>CRG 732-11Y-010M-0Y
53	Gomal -08	V63	2008	ATTILA<br>CM 85836-4Y-0M-0Y-14M-0Y-5M-0Y-1SJ-0Y
54	Hashim -08	V64	2008	JUP/ALD‟S‟//KLT‟S‟/3/VEE‟S‟/6/BEZ//0APTOB/8156/4/ON/3/<br>6*TH/KF//6* LEE/KF/5

				ICW91-0321-2AP-0TS-1AP-2AP-0L-0AP
55	TD - 1	V65	2004	MAI'S'/NORTEN065 /H68<br>CM 59695
56	SKD - 1	V66	2006	HD-2329<br>PAU-ACC-3079
57	Imdad - 05	V67	2006	CHIL/2*STAR<br>CM112793-0TOPY-8M-020-010M-3Y-010M-10Y
58	Pir Sabak - 2008	V68	2008	KAUZ/PASTOR<br>CMSS03B00025S-48Y-010M-010Y-010M-4Y-0M
59	Jauhar -78	V69	1978	Nayab* PJ62/GB55//GB56/3/TZPP/NAI60MUTANT NF 600 RADS
60	NIA - Amber	V70	2010	VEE//5 'S'/SARA//SOGHAT90
61	Punjab -2011	V73	2011	ALTAR84/AE.SQUARROSA.(219)01//SERI<br>CMBW91Y008925-8Y-11KBY-2KBY-010M-9Y-3M-0Y-05Y
62	Khirman	V74	2006	ULC/PVN//TAN/3/BUC
63	Millat - 2011	V75	2011	CHENAB2000/INQ.91
64	Janbaz-10	V76	2010	Gen*2//Buc/Filk/3/Buchin
65	Dharabi-11	V77	2011	HXL7573/2*BAU//PASTOR<br>CMSS97Y03676S-040Y-050M-040SY--030M-21SY-010M-0Y-0SY
66	NARC - 2011	V78	2011	OASIS/SKAUZ//4*BC /3/2*PASTOR<br>CMSS00Y01881T-050M-030Y-030M-030WGY-33M-0Y-01D
67	Tajban-10	V79	2010	W3918A/JUP
68	Shahkar -13	V81	2013	CMH84.3379/ CMH78.578//MILAN<br>CMSS93Y006285-7Y-010Y-010M-010Y-010M-0Y-3KBY-0KBY
69	Pirsabak -13	V82	2013	CS/TH.SC//3*PVN/3/MIRLO/BUC/4/MILAN/5/TILHI<br>CMSS97M04005T-040Y-020Y-030M-020Y-040M-28Y-3M-0Y
70	Atta Habib	V83	2010	INQILAB 91*2/TUKURU<br>CGSS99B00015F-099Y-099M-099Y-31Y- OB
71	Seren-2007	V84	2007	PBW343*2/KUKUN<br>CGSS99B00041F-099Y-099M-099Y-099M-34Y-OB
72	Hamal - Faqir	V85	2012	LFN/1158.57//PRL/3/HAHN/4/KAUZ/5/KAUZ<br>CMB89Y1044-0T0PM-8Y-010M-020B-0NPL-010Y-3M-015Y-0Y
73	Pakistan - 2013	V86	2013	PMEX94.27.1.20/3/SOKOLL//ATTIALA/3*BCM<br>PTSS02B00132T-0TOPY-0B-0Y-OB-38Y-0M-OSY-OID
74	NIA - Sunder	V87	2011	Sarsabz/Sunco*2`
75	NIA -Saarang	V88	2012	SHA4/WEAVER/SKAUZ*2/SRMA
76	NIFA - Lalma	V89	2012	PASTOR/3/ALTAR84/AESQ(TAUS)//OPATA(SOKOLL)<br>CMSS97M00316S-OP20M-OP20Y-43M-0Y
77	Chenab -79	V91	1979	PB76/CH70
78	Pirsabak - 85	V92	1986	KVZ/BUHO/BB<br>CM33027F-1SM-4Y-4M-2Y-1M-1Y-0M
79	Pakhtoonkhwa -15	V93	2015	WBLLI*2/4/YACO/PBW65/3/KAUZ*2/TRAP//KAUZ<br>CGSS01Y00054T-099M-099Y-099M-099M-20Y-0B
80	Pirsabak - 15	V94	2015	MILLAN/S87230//BABAX<br>CMSS97M03689T-040Y-030M-020Y-030M-015Y-030M-3Y-1M-0Y
81	Zincol-16	V95	2016	CROC_AE.SQUARROSA(210)//INQILAB91*2/KUKUNA /3/ PBW343* 2/ KUKUNA<br>CMSA06M00195-099Y-099Y-099M-9M-0Y-7B-0Y-0ID
82	Borolug -16	V96	2016	RFUG99RE 09/10 CIMMYT Selection#08 (2009-10)<br>Ptss02b00015S-0Y-0B-0Y-1Y-0M-0SY-0ID
83	Ujala -15	V97	2015	KIRITATI/4/2*//WEAVER/TSL//WEAVER/3/WEAVER

84	IBGE-Ghaneemat-15	V98	2015	CROC-1/AE.SQUARROSA(205)//KAU2/3/ATTILA CMSS93Y01031S-13Y-5KBY-010M-010Y-5M-0KBY-0M-9KBY-…
85	Shalakot -13	V99	2014	TRACHA'S'//CMH76-252/PVN'S'
86	NIFA - Aman	V100	2017	PRL/2*PASTOR//PBW343*2/KUKUNA/3/ROLY07 29CMSS04B00025T-0TOPY-09922TM-099Y-8WGY-0B
87	NIFA - Insaf	V101	2015	Tatara/Inqilab-91
88	Gold - 16	V102	2016	PR-32(BAU)/INQ-91
89	Jouhar -16	V103	2016	KAUZ/PASTOR//V.3009 BR.4896-1B-2B-1B-0B
90	Ihsan-16	V104	2016	PASTOR/3/ALTAR84/AESQ//OPATA
91	Fathejang -16	V105	2016	ERA F2000/4/FONG CHAN#3/TRT'S'//VEE#9/3/COOK/VEE'S'// DOVE'S'/SERI FJ2003-61-0F-53F-46F-43F-16F-8F-0F
92	NN - Gandum - 1-2016	V106	2016	Chirya-3/Opata//2*Parula/3/Rohtas-90
93	Ujala -16	V117	2016	KRITATI/4/2*// WEAVER/TSL //WEAVER/3/WEAVER
94	Anaj-2017	V118	2017	BABAX/LR43//BABAX/6/MOR/VEE#5//DUCULA/3/DUCULA/4/MILAN/5/BAU/M ILAN/7/SKAUZ/BAV92 CMSS05Y00558T-099TOPM-099Y-099M-099Y-099ZTM-7WGY-0B
95	AARI-2011	V120	2011	SH.88/90A204//MH.97
96	Galaxy -2013	V121	2013	PB96/87094/MH97 PB30332-0A-0A-0A-9A-27A
97	Gomal-08	V122	2007	ATTILA CM 85836-4Y-0M-0Y-14M-0Y-5M-0Y-1SJ-0Y
98	Shafaq - 2006	V123	2006	LU 26/HD2179//2* INQ-91 PB 28633P-2A-6A-0A
99	Sehar-2006	V124	2006	CHIL/2*STAR/4/BOW/ CROW//BUC/PVN/3/ 2*VEE#10 CMSS9Y00645-100Y-200M-17Y-10M-0Y-0P-PAK
100	Faisalabad-2008	V126	2008	PBW65/2*PASTOR CGSS97Y000367-099TOPB-067Y-099M-099Y-099B-16Y-0B

Supplementary Table S1. Parentage of wheat varieties selected to study the correlation of root system architecture with reference to grain Zn content.

Table. S2 Mean values of selected 12 wheat varieties root system architecture

Varieties	Number	PRL	LRN	TLRL	TRL	LRD	Zn Conc
Punjab-11	E1	16.54	2	83.45	99.99	0.14	17.01
Fatehjang-16	E2	19.16	2	84.19	103.35	0.10	15.75
Zincol-16	E3	18.29	4	89.61	107.90	0.09	22.59
Pirsbak-13	E4	17.15	3	81.04	98.19	0.21	5.22
NIA-Sunder	E5	17.09	3	66.48	83.56	0.16	5.49
Shakar-13	E6	16.66	2	72.41	89.07	0.16	5.31
TD-01	E7	2.04	2	5.61	7.65	0.74	14.13
Jauhar-16	E8	2.35	5	5.66	8.02	0.71	14.31
Mairaj-08	E9	2.33	2	6.95	9.28	2.14	13.86
Fsd-08	E10	1.00	3	4.78	5.78	3.12	5.04
Aas-09	E11	1.60	4	6.09	7.69	1.67	4.86
BARS-09	E12	1.28	4	4.72	6.00	1.99	4.95

Supplementary Table. S2 Attributes related to RSA and grain Zn conc of selected 12 wheat varieties from Study 1(rhizobox study). PRL(Primary root length), LRN (lateral root number), TLRL (Total lateral root length), TRL (total root length), LRD (lateral root density), Zn conc (Zn concentration in grain).

Photo. S1 Roots images of selected 12 wheat varieties taken out from the rhizobox.

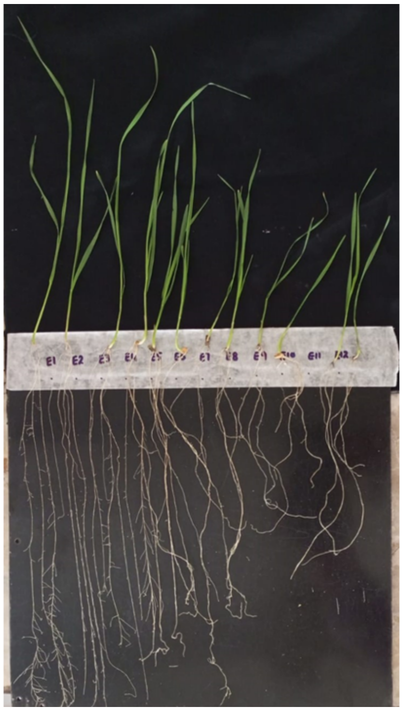

Supplementary Photo. S1 E1-E3 have higher Zn content of grains and vigorous root system architecture, E4-E6 have lower Zn content of grains and vigorous root system architecture, E7-E9 have Higher Zn content of grains and weaker root system architecture, E10-E12 have lower Zn content of grains and weaker root system architecture.
